# Three-dimensional visualization of heart-wide myocardial architecture and vascular network simultaneously at single-cell resolution

**DOI:** 10.3389/fcvm.2022.945198

**Published:** 2022-08-04

**Authors:** Jianwei Chen, Guangcai Liu, Wen Sun, Yuanfang Zheng, Jing Jin, Siqi Chen, Jing Yuan, Hui Gong, Qingming Luo, Xiaoquan Yang

**Affiliations:** ^1^Britton Chance Center for Biomedical Photonics, Wuhan National Laboratory for Optoelectronics, MoE Key Laboratory for Biomedical Photonics, Huazhong University of Science and Technology, Wuhan, China; ^2^HUST-Suzhou Institute for Brainsmatics, Jiangsu Industrial Technology Research Institute (JITRI), Suzhou, China; ^3^CAS Center for Excellence in Brain Science and Intelligence Technology, Chinese Academy of Science, Shanghai, China; ^4^School of Biomedical Engineering, Hainan University, Haikou, China

**Keywords:** whole heart, 3D imaging, single-cell resolution, vasculature, cardiomyocytes

## Abstract

Obtaining various structures of the entire mature heart at single-cell resolution is highly desired in cardiac studies; however, effective methodologies are still lacking. Here, we propose a pipeline for labeling and imaging myocardial and vascular structures. In this pipeline, the myocardium is counterstained using fluorescent dyes and the cardiovasculature is labeled using transgenic markers. High-definition dual-color fluorescence micro-optical sectioning tomography is used to perform heart-wide tissue imaging, enabling the acquisition of whole-heart data at a voxel resolution of 0.32 × 0.32 × 1 μm^3^. Obtained structural data demonstrated the superiority of the pipeline. In particular, the three-dimensional morphology and spatial arrangement of reconstructed cardiomyocytes were revealed, and high-resolution vascular data helped determine differences in the features of endothelial cells and complex coiled capillaries. Our pipeline can be used in cardiac studies for examining the structures of the entire heart at the single-cell level.

## Introduction

The heart is the key organ in the circulatory system responsible for pumping blood throughout the body. Myocardial and cardiovascular tissues are the main components of the heart and assist the heart in functioning properly. In particular, the cardiac vasculature is the main venue for the exchange of oxygen and carbon dioxide in the blood as well as nutrients and waste. Moreover, through the contraction, cardiomyocytes provide pressure for blood pumping. Thus, abnormalities in myocardial and vascular structures may affect the functions of the heart, causing cardiomyopathies and cardiovascular diseases (1). Atherosclerosis is a cardiovascular disease with high incidence and is characterized by thickening of the arterial wall (2). Previous studies have mainly focused on arterial abnormalities. However, less attention has been paid to capillaries that are involved in microcirculation and are critical in the cardiovascular system (3). Hence, the complete vasculature network should be visualized at the capillary level in cardiovascular disease studies. Hypertrophic cardiomyopathy has a typical pathology of myocyte disarray; however, the heart-wide distribution of such structural abnormalities is not completely clear (4). Therefore, imaging of the entire myocardium at the single-cell resolution can help locate lesions. Moreover, because both myocardial and vascular abnormalities are involved in some microcirculatory diseases, the interaction between the myocardium and intramyocardial vessels affects blood flow (5, 6). Thus, the simultaneous acquisition of myocardial and vascular images can reveal their spatial correlation (7). Heart-wide fine structures, especially the myocardium at the single-cell scale and the cardiac vasculature at the capillary level, must be examined in research on cardiac mechanisms and diseases.

Substantial efforts have been made to visualize the heart-wide structure. Microcomputed tomography and magnetic resonance imaging have been mostly used in traditional cardiac imaging and provide a basis for gross structural studies and clinical disease diagnoses (8, 9). However, because these technologies are limited by an intrinsic resolution of approximately 10 μm, they fail to reveal the fine structure of cardiomyocytes and capillaries. An optical microscope with submicron spatial resolution is a suitable tool for measurement. However, an optical microscope fails to cover the entire heart because of high optical scattering caused by the complex refractive index of biological tissues. Therefore, optical scattering is the main obstacle that must be overcome. Various optical clearing technologies have been used to make heart tissue transparent (10, 11). After optical clearing, a light-sheet microscope can be used for imaging the entire heart at micron-scale spatial resolution. However, optical clearing cannot fundamentally solve the problem of compositional differences among heart tissues, resulting in poorer images at greater imaging depths (12). To date, methods for the three-dimensional (3D) imaging of cardiomyocytes and cardiovasculature in the intact mature heart at submicron resolution are not available.

Mechanical sectioning of tissues, which is widely used in histological studies, is another strategy for overcoming optical scattering (13, 14). The tissue is embedded and cut using a microtome into slices with a thickness of several microns. Subsequently, an optical microscope can be used for the imaging of sectioned tissues without interference from optical scattering. However, this method provides only two-dimensional (2D) information on the tissue. In the last decade, several strategies combining a microtome and block-face imaging have been proposed for obtaining 3D structural information on the entire organ (15–18), including fluorescence micro-optical sectioning tomography (fMOST). Scholars have successfully used fMOST to examine the comprehensive anatomy of the brain, including the cell architecture, neural circuits, and vascular networks (19–21). Although this promising technology can be applied in cardiac imaging, some challenges still need to be addressed. First, the heart should be properly labeled and embedded to ensure optical contrast and adequate hardness, respectively. Second, the optical microscope and microtome should be well aligned to ensure high imaging quality. Third, the acquired images of cardiomyocytes and cardiovasculature should be processed for further analysis.

In this study, we established a technical pipeline for labeling and imaging cardiac architecture and networks within a given mouse heart. We labeled cardiomyocytes and cardiovasculature by using a fluorescent dye and transgenic marker, respectively. Subsequently, we acquired the images of these two structures at a voxel resolution of 0.32 × 0.32 × 1 μm^3^ through the latest fMOST approach, namely high-definition dual-color fMOST (HD-dfMOST). We present these two structures individually to reveal the myocardial configuration and vascular network. In addition, we show these two structures simultaneously to display their spatial correlation. Furthermore, we visualized curvature changes in the vascular wall by using 3D traced and reconstructed vascular data. The results demonstrated the feasibility of our pipeline and the superiority of the single-cell resolved 3D cardiac data set.

## Materials and methods

### Overview of the pipeline for 3D imaging of the whole heart at single-cell resolution

**Figure 1:**
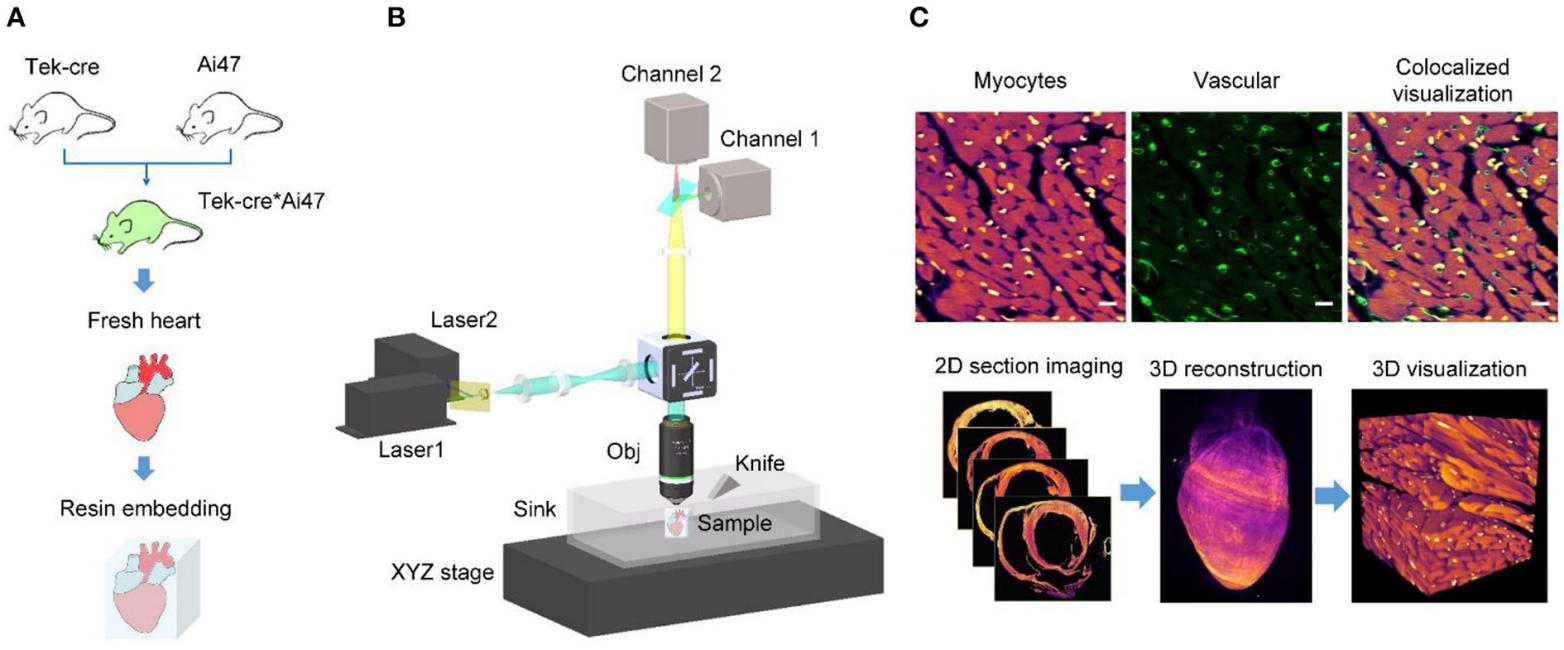
Single-cell-level heart-wide data acquisition pipeline. **(A)** Whole-heart sample preparation. We produced vascular fluorescent-labeled transgenic mice (Tek-cre*Ai47) by crossing two transgenic mice (Tek-cre and Ai47), obtained their fresh heart tissues, and embedded them into HM20, a stiff resin. **(B)** System setup of HD-dfMOST. The system is composed of a line-illumination modulation (LiMo) microscope and a microtome. (Obj, objective lens; sCMOS, scientific complementary metal oxide semiconductors). **(C)** Cardiac fMOST data were processed and are shown in 2D and 3D.

We developed a three-step pipeline for tissue preparation, heart-wide imaging, and data processing, respectively (Figure 1). Briefly, in Step 1, the heart tissue was labeled with fluorescent proteins and embedded in resin to prepare it for imaging and cutting. In Step 2, the heart tissue was sequentially imaged and cut under the guidance of HD-dfMOST until data for the whole heart had been acquired. In Step 3, the myocardial and vascular data were processed and observed at single-cell resolution.

In Step 1, we prepared the heart tissue for subsequent fluorescence imaging and tissue cutting (Figure 1A). To genetically label blood vessels, we crossed Tek-Cre mice with Ai47 mice (Tek-Cre:Ai47 mice), wherein we labeled endothelial cells present on the microvascular wall with green fluorescent protein [GFP; (22)]. We used the fluorescent dye propidium iodide (PI) for the real-time staining of myocardial architecture during imaging (17). Furthermore, we embedded the heart tissue into HM20, a stiff resin, for thin tissue sectioning and fluorescent protein expression imaging (23).

In Step 2, we performed heart-wide *in-situ* imaging through HD-dfMOST (Figure 1B). Details of the HD-dfMOST system were reported in our previous study (18). The system comprised a line-illumination modulation (LiMo) microscope and a custom-built microtome. In the LiMo microscope, two lasers with wavelengths of 488 and 561 nm, respectively, were coupled and modulated as a line beam through a cylindrical lens and then focused on the sample surface through an objective lens to excite the GFP and PI. Fluorescence signals were collected by the objective lens and a tube lens and subsequently detected by two scientific complementary metal oxide semiconductor (sCMOS) cameras (i.e., channels 1 and 2). The microtome consisted of a fixed diamond knife and a three-axis linear motorized stage to cut the stiff resin into thin sections with a high degree of flatness. Specifically, every time, a shallow layer of a 2-μm-thick tissue was imaged using the LiMo microscope, in which a 660-μm-wide and 1-μm-thick strip stack was imaged through line scanning back and forth. Subsequently, the tissue was aligned with and moved toward the microtome at a feed speed of 5 mm/s to remove the imaged surface, exposing the new block face for myocardial architecture staining. The sample was then lifted for the next round of tissue imaging and cutting. These steps were repeated until the whole heart had been imaged. We obtained the 3D data set of the entire heart at a consistent voxel resolution of 0.32 × 0.32 × 1 μm^3^.

In Step 3, we processed the acquired data and obtained 2D and 3D high-resolution heart-wide myocardial and vascular images (Figure 1C). The data acquired using the LiMo microscope were in the form of strip images. We stitched these strips and removed their neighboring overlap to obtain complete section images. The original imaging data had a voxel size of 0.32 × 0.32 × 1 μm^3^. To facilitate the subsequent processing and analysis of terabyte-scale whole-heart volumetric images, the original data sets were reformatted from TIFF to TDat at multilevel resolution (24). For 2D images, myocardial and vascular structures were colored in fuchsia and green, respectively, in ImageJ software (version 1.51, National Institutes of Health, Bethesda, MD, USA). Typically, the myocardial image is displayed with a thickness of 1 μm to present the details of cardiomyocytes, whereas the vascular image is displayed with a thickness of hundreds of microns to demonstrate the connectivity of the network. Morphological parameters, such as nucleus size and vessel diameter, were quantitatively analyzed using ImageJ software. In the detailed operation, we defined the distance of each pixel in the set scale and the start and end points of specific features and measured the feature size. The depth coding of a partial capillary network (**Figure 3D**) was generated using a depth coding algorithm and processed using Matlab software (version R2017a, The MathWorks Inc., Natick, MA, USA). Two structures were simultaneously displayed using ImageJ software by merging two-channel images. For 3D images, the whole heart was reconstructed three dimensionally by stacking sequential 2D images in Amira software (version 6.11, FEI, Mérignac Cedex, France). The data were down-sampled to a voxel size of 10 × 10 × 10 μm^3^ for displaying the whole heart because the original whole-heart data set had a size of approximately 7 TB. Thus, the existing computer memory and image software were unable to process such a large amount of data directly. Partial raw data from the regions of interest were rebuilt using Amira software with a voxel size of 0.32 × 0.32 × 1 μm^3^. Furthermore, a partial vascular network was traced using the down-sampled vascular data with a voxel size of 10 × 10 × 10 μm^3^ in Amira software (**Figures 4F**, **5**). First, we located the aorta on the basis of the original axial image (**Figure 3A**). The aorta could be easily identified because it had an obvious hollow shape and was located in the middle of the axial image. Next, we continuously traced the vascular branch by using consecutive sequential images. We segmented the outline of blood vessels in 2D mode by using the segmentation editor in Amira software. In particular, we set the segmentation threshold to automatically obtain the foreground of blood vessels and then examined automatically segmented images one by one and manually labeled inaccurate segmentation. After the completion of segmentation, we used the SurfaceGen module of Amira software to automatically extract the surface model of the vessel contour and perform smoothing of the 3D model in Amira. Furthermore, we used the GetCurvature function in Amira to visualize the curvature of blood vessels (**Figure 5**).

### Animals

Tek-Cre mice were obtained from Jackson Laboratory (Stock No. 008863, Bar Harbor, ME, USA). Ai47 mice were kindly donated by Dr. Hongkui Zeng. Ai47 mice were crossed with Tek-Cre mice, and a double-positive mouse (8 weeks old) was used in the present study. Because the Ai47 mouse expresses only GFP and the Tek mouse expresses no fluorescent reporter, the crossed mouse expresses only GFP (19). All mice were kept under a 12-h light/dark cycle with food and water provided *ad libitum*.

### Tissue preparation

Mice were deeply anesthetized with 1% sodium pentobarbital solution (1% wt/vol) and subsequently perfused with 0.01 M phosphate-buffered saline (PBS, Sigma-Aldrich Inc., St Louis, MO, USA) to flush blood vessels, followed by perfusion with 4% (1% wt/vol) paraformaldehyde (Sigma-Aldrich Inc.) and 2.5% sucrose in 0.01 M PBS for fixation. The hearts were excised and fixed in 4% paraformaldehyde at 4°C for 24 h. After fixation, the intact hearts were rinsed overnight at 4°C in 0.01 M PBS solution.

### Embedding method

To embed a whole mouse heart, the intact heart was dehydrated in graded ethanol series (50, 75, and 95% ethanol, changing from one concentration to the next in 1, 2, and 2 h, respectively; then, the samples were incubated in 100% ethanol for 1, 2, and 2 h each time, respectively, and the ethanol solution was changed three times). Subsequently, the sample was infiltrated in a Lowicryl HM20 resin kit (Electron Microscopy Sciences, Hatfield, PA): 50% and 75% for 2 h each and 100% for 2, 24, and 24 h, respectively. Finally, the whole heart was embedded in a gelatin capsule filled with HM20 and polymerized at 38°C for 24 h, at 45°C for 8 h, and at 52°C for 6 h.

### Mouse heart imaging

To obtain vascular and myocardial information on the mouse heart, the sample was placed on the HD-dfMOST system for sectioning and imaging. The whole sample was immersed in a water sink containing PI solution for real-time staining of the myocardial architecture. Whole-heart imaging was performed in a water bath. In our experiment, the sample was imaged at a voxel resolution of 0.32 × 0.32 × 1.0 μm^3^. The heart sample colabeled with myocardial architecture and blood vessels was imaged through two channels (GFP and PI, respectively).

The following devices were used in the HD-dfMOST system (Figure 1B): lasers (488 and 561 nm, Cobolt, Sweden), lenses (L1, AC050-008-A-ML, f 7.5 mm; L2, AC254-250-A, f 250 mm; L3, TL, AC254-125-A-ML, f 125 mm, Thorlabs, USA), a cylindrical lens (ACY254-100-A, f 100 mm, Thorlabs, USA), an objective lens (XLUMPLFLN 20XW, 1.0 NA, Olympus, Japan), dichroic mirrors (ZT488/561rpc Chroma USA), excitation filters (ZET488/561 m, Chroma, USA), sCOMS cameras (ORCA-flash4.0 V3, Hamamatsu, Japan), and an XYZ linear stage (x-axis XML210, y-axis XMS100, z-axis GTS30V, Newport, USA).

### Depth-coding algorithm

The algorithm for capillaries depth-coding is as follows:

**Input:** Image numbers k, Binarization threshold

**Output:** Depth-coded images

**for** *n* = *1 to k* **do**

**repeat**

Image binarization

Linearize the color bar according to the number of images

Assign a single color to each image according to the order of the color bar and the order of images correspondingly

Stack these colored images sequentially

**until** all the images have been proceed

**end for**

## Results

### Display of heart-wide myocardial structures at single-cell resolution

**Figure 2:**
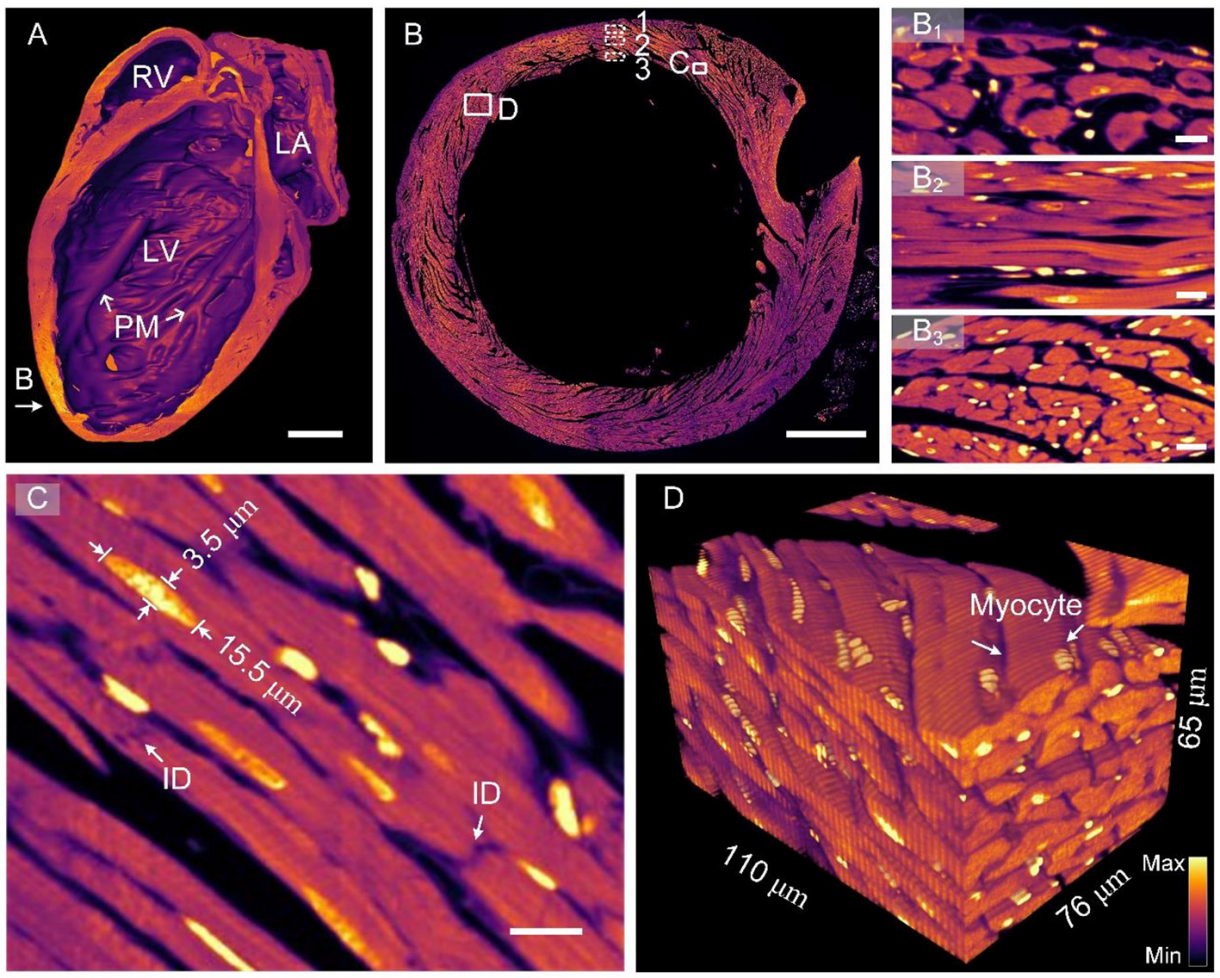
Images of heart-wide myocardial architecture at single-cell resolution. **(A)** Sagittal section view of reconstructed whole-heart myocardial data. LA, left atrium; LV, left ventricle; RV, right ventricle; PM, papillary muscle. Scale bar: 1 μm. **(B)** Original image of an axial section of the heart. **(B**_**1**_**–B**_**3**_**)** display the cardiac muscle cells of the ventricular wall from the outside layer (epicardium), middle layer (myocardium), and inside layer (endocardium). Scale bars: 500 and 10 μm, respectively. **(C)** Enlarged view of the corresponding rectangle in **(B)**. ID, intercalated disc. Scale bar: 10 μm. **(D)** A reconstructed myocardial data block with a single resolvable myocyte.

Figure 2 presents myocardial data collected through HD-dfMOST. The myocardial fluorescent signal was excited by a 561-nm laser, and 9,972 sagittal sections were collected with a data size of 6.09 TB and colored in fuchsia. Figure 2A presents the 3D rendering of the half heart in a coronal view. The chambers of the heart are displayed, including the left ventricle (LV), right ventricle (RV), and left atrium (LA). The ventricular wall of the LV was thicker than that of the RV, which is consistent with the gross anatomy (25). In particular, papillary muscle was observed in the LV, indicating that resin embedding provides adequate physical support to the hollow organ. Even the dangling PM tissue was well preserved after ultrathin tissue cutting. Figure 2B presents the raw data of an axial image with a voxel resolution of 0.32 × 0.32 × 1.0 μm^3^, and Figure 2A indicates the position of the section. Figures 2B_1_–B_3_ show regions located on the epicardium, myocardium, and endocardium of the ventricular wall, respectively (Figure 2B). The cardiomyocytes were differently oriented in these images. The subepicardial muscle fibers were oriented vertically downward, the medial muscle fibers were horizontally arranged in a ring, and the subendocardial muscle fibers were vertically oriented. These findings indicated that the arrangement of ventricular myocardial fibers was divided into three layers, which is consistent with the results of a previous study (26). Fine structures of cardiomyocytes are presented in the enlarged Figure 2C, corresponding to the white rectangle in Figure 2B. The nuclei were located in the middle of cardiomyocytes. The measured nucleus had an elliptical shape with a major axis of 15.5 μm and a minor axis of 3.5 μm. Moreover, intercalated discs (IDs) connecting cardiomyocytes were identified; they are indicated by white arrows. The IDs appear dark because PI mainly binds to genetic molecules such as DNA and RNA and the IDs contain no genetic molecules. These cell junctions normally have a jagged shape and own a feature size at the submicron level (27); the high resolution enabled us to identify them. Figure 2D presents a 3D reconstruction of cardiomyocytes from 65 raw images corresponding to the selected area in Figure 2B. This image allows us to three-dimensionally visualize the morphology and orientation of cardiomyocytes. Each cardiomyocyte appeared cylindrical in the 3D image. In addition, the spatial arrangement of cardiomyocytes was displayed; the local group of cardiac muscle cells had a similar trend, which is consistent with the traditional anatomical model diagram (28). Supplementary Video 1 presents rendered myocardial data, more intuitively showing the morphology and orientation of myocardial assemble. The results indicate that the proposed pipeline can be used to microscopically identify cardiomyocytes at the single-cell level and visualize them three dimensionally in combination with PI counterstaining and HD-dfMOST imaging.

### Exhibition of the whole-heart comprehensive vascular network at the capillary level

**Figure 3:**
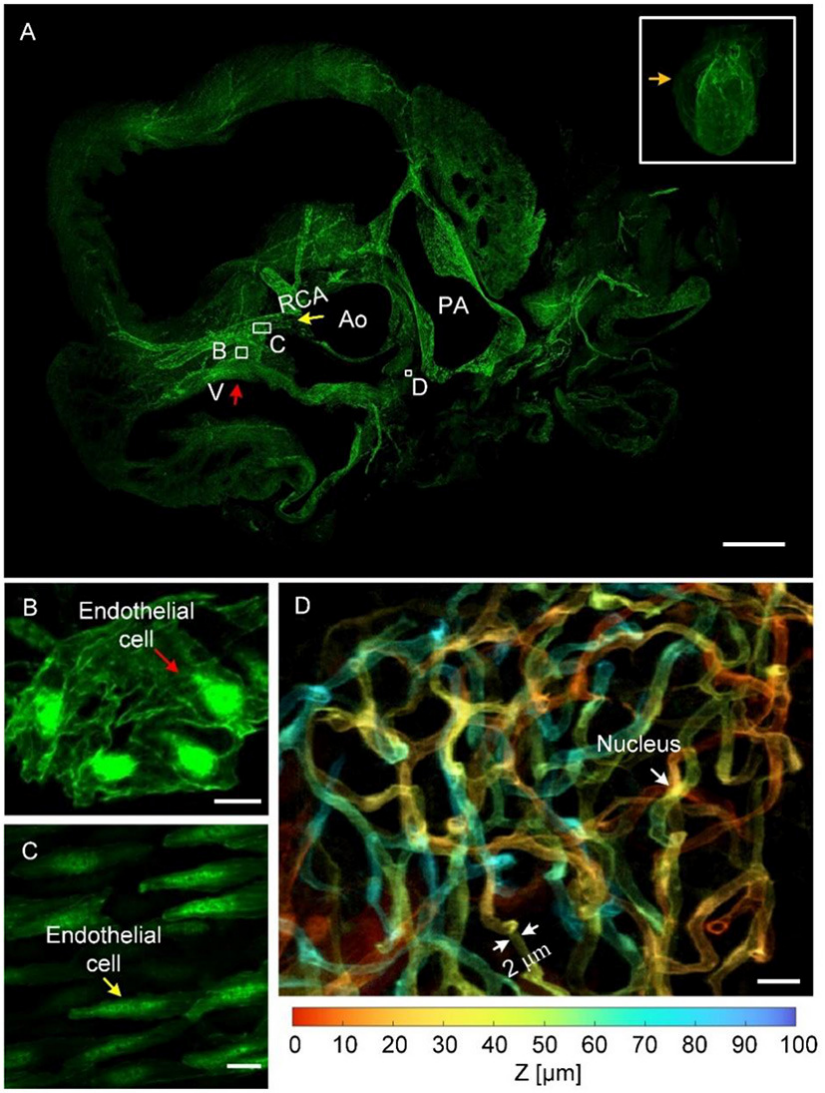
Images of the whole-heart vascular network at the capillary level. **(A)** The 3D rendering of whole-heart vascular data (top right) and the MIP generated from raw axial vascular images with a thickness of 300 μm. V, vein; RCA, right coronary artery; Ao, aorta; PA, pulmonary artery. Scale bar: 500 μm. **(B,C)** Scaled images corresponding to the white rectangles in **(A)**, with an MIP of 100-μm-thick raw images, displaying endothelial cells in the vein and RCA, respectively. Scale bars: 10 μm. **(D)** An enlarged view of the area indicating a depth-coded 100-μm-thick MIP image **(A)**, showing a partial capillary network with depth information. Scale bar: 10 μm.

Figure 3 presents cardiovascular data imaged through HD-dfMOST. The vascular signal was excited using a 488-nm laser, and the data had a size of 0.96 TB. The 3D rendering of the whole-heart vascular network is shown in the top right rectangular area of Figure 3A. The 3D contour of the heart was observed as a cone shape through the background signal. The maximum intensity projection (MIP) of 300 selected raw axial images is shown in Figure 3A, and the location of the images is indicated by an orange arrow. The right coronary artery (RCA) extending from the aorta (Ao) and crossing the vein (V) was observed in the MIP image. The endothelial cells of the vein and the RCA are shown in the enlarged Figures 3B,C through the stacking of 10 and 100 raw images, respectively. Every endothelial cell, even their nuclei, could be observed due to the submicron imaging resolution of HD-dfMOST. The endothelial cells of the vein appeared in polygons with spherical nuclei located in the middle (Figure 3B). The cells of the RCA appeared in fusiform with nuclei of similar shape (Figure 3C). The morphological heterogeneity of endothelial cells was reported in a previous study (29) but has rarely been studied through *in-situ* 3D optical imaging. Partially complicated capillaries closed to the pulmonary artery are shown in the enlarged Figure 3D, which was obtained by stacking 100 raw images and corresponds to the white rectangle in Figure 3A. Multiple winding branches in this 100-μm-thick section were noted. Here, we coded the depth information of capillaries into different colors, which helped us visualize their connectivity and spatial relationship. In addition, by using HD-dfMOST, we could resolve capillaries with a diameter of at least 2 μm. In particular, the nuclei of the capillaries appeared as long bright stripes, demonstrating the high resolution of the imaging system. Supplementary Video 2 further presents rendered cardiovascular data, intuitively showing the cardiovascular network and the complex three-dimensional spatial winding of capillaries. These results confirm that the proposed pipeline is capable of labeling and imaging whole-heart vessels at the capillary level.

### Simultaneous presentation of myocardial and vascular structures in a given heart

**Figure 4:**
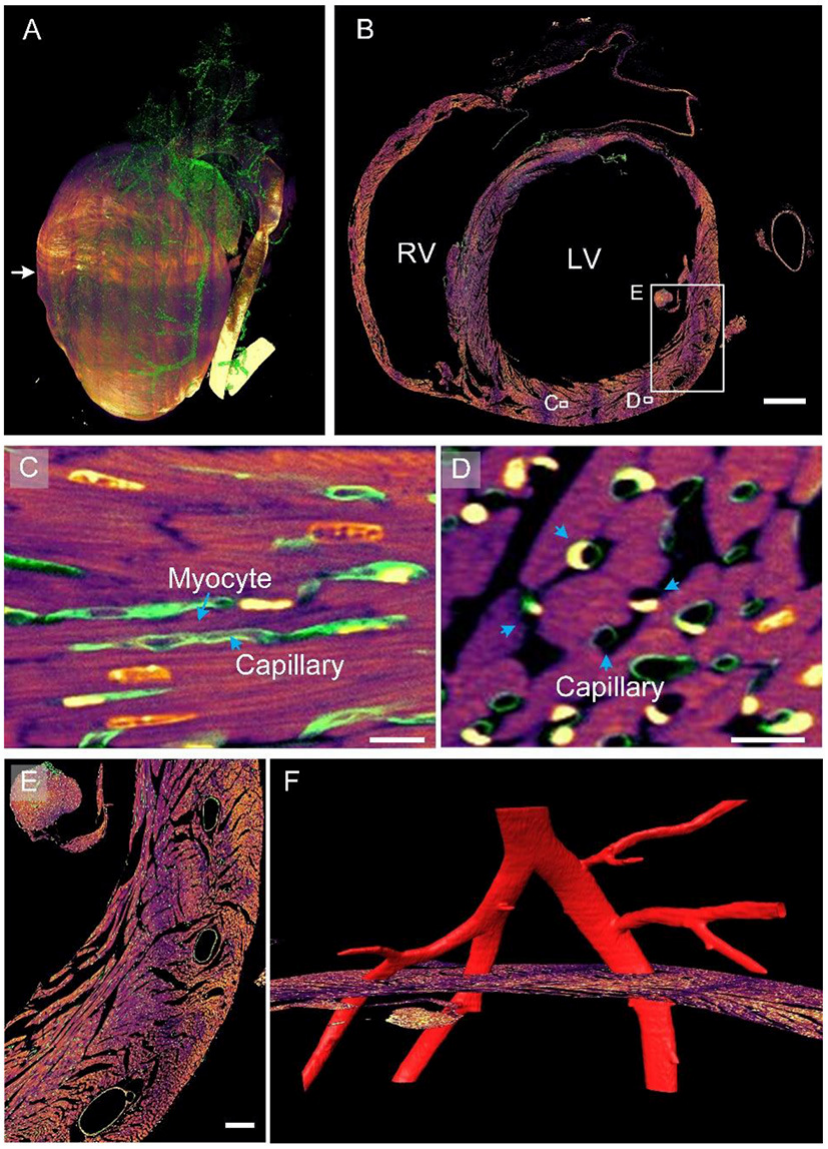
Myocardial and vascular data of the entire heart within a given mouse. **(A)** The 3D rendering of heart-wide myocardial and vascular data. The myocardial structure is in fuchsia and the vascular structure in green. **(B)** A selected axial section in the middle of the heart. LV, left ventricle; RV, right ventricle. Scale bar: 500 μm. **(C,D)** Enlarged views of corresponding white boxes in **(B)** revealing the spatial correlation between cardiomyocytes and capillaries. Scale bars: 10 μm. **(E,F)** Enlarged image of the white square in **(B)**. Scale bar: 100 μm.

On the basis of the dual-structural labeling and dual-color imaging achieved using the proposed pipeline, we present myocardial and vascular data of a heart colocalized in one coordinate. The 3D rendering of the myocardial and vascular structures of the whole heart is shown in Figure 4A, where the myocardial structures are colored fuchsia and the vascular structures green. The heart is a muscular organ that mainly consists of myocardial tissues, as indicated by the image mainly appearing fuchsia in color. The blood vessels were continuously labeled and completely observed in the image. An original axial image located in the middle of the heart at a resolution of 0.32 × 0.32 × 1.0 μm^3^ is displayed in Figure 4B. Capillaries were distributed between cardiomyocytes, as shown in the enlarged Figure 4C, corresponding to the white rectangle in Figure 4B. Small vessels were located right in the gaps in the myocardium, indicating that the two-channel data were co-located. The capillaries and cardiomyocytes had the same orientation. The observations are consistent with the cardiac mechanism indicating that capillaries supply nutrients and oxygen to cardiomyocytes (30). Both cardiomyocytes and capillaries appeared as quasi-circular cross-sections with a staggered arrangement (Figure 4D), indicating that these two structures were spatially close. This means that cardiomyocytes can provide pressure for pumping blood through muscle contraction (30). Supplementary Video 3 further presents rendered co-localized myocardial and vascular data stereo showing the spatial distribution of myocardial and vascular structures. Three hollow elliptical structures were distributed on the outer edge of the ventricular wall (Figure 4E), indicating that three blood vessels may travel through the ventricular wall. We reconstructed the vascular data three dimensionally and observed that an artery was divided into three branches passing through the three cavities (Figure 4F), confirming our assumption. This example indicates that the pipeline can be used to examine the spatial correlation between cardiomyocytes and vessels in 2D or 3D.

### 3D display of the continuous blood vessel network

**Figure 5:**
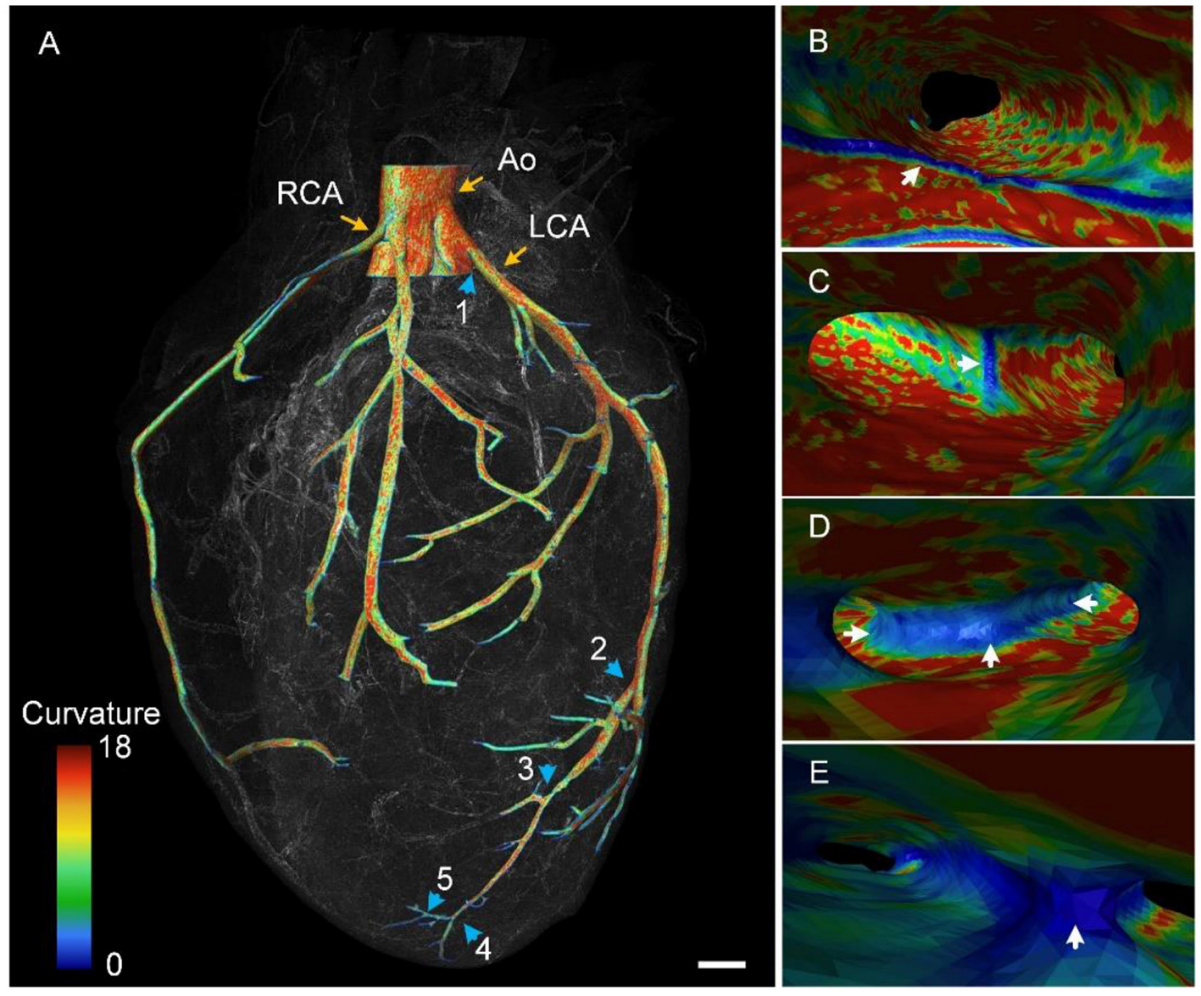
Color-coded vascular network based on the curvature of the vascular wall. **(A)** The 3D rendering of the reconstructed vascular network. Blue arrows point out five branches along the LCA. Ao, aorta; LCA, left coronary artery; RCA, right coronary artery. **(B–E)** Endoscopic perspective of corresponding branches 1–4 in **(A)** with colors representing the curvature difference. **(B)** is the division of the Ao and LCA.

We reconstructed a partial cardiovascular network in 3D, starting from the aorta (Figure 5A). The aorta divided into two branches, namely the left coronary artery (LCA) and RCA, as shown in the reconstructed result (Figure 5A). The RCA divided into two equal dominated branches, whereas the LCA diverged after some distance, which is consistent with the findings of a previous study (25). Here, we mainly traced the branches of the LCA. At least five branches were identified along the trunk from the aorta to the apex of the heart (as indicated by blue arrows). In addition, we obtained the curvature of vessels in accordance with labeled contours. The difference in curvature of the vessels is qualitatively represented using colors in Figure 5A. As indicated by the color bar, the maximum curvature is presented in red, whereas the minimum curvature is presented in blue. The overall trend of the curvature of the outer contour was a gradual decrease with increasing distance of the branch from the aorta. In addition, we visualized these vessels in an endoscopic view, observing detailed curvature changes from the inner wall of the blood vessel. The inner walls of branches 1–4 are presented in Figures 5B–E, respectively. The inner wall of the LCA had larger curvature because the wall mostly appeared in red (Figure 5B). However, the junction of the aorta and LCA appeared in blue, as indicated by the white arrow, indicating smaller curvature. The smaller curvature may be due to such a shape providing a smooth transition between vascular branches, avoiding sudden and considerable blood flow pressure at these locations. Similar morphological characteristics are presented in Figures 5C–E. We observed that the small curvature area became larger with a decrease in the vessel diameter, as indicated by white arrows in Figures 5C,E. This trend was possibly caused by a decrease in blood pressure with a decrease in the vessel size (31). In summary, the proposed pipeline provided multiple views of 3D cardiac microstructures and thus can be used for comprehensively studying these structures and functions.

## Discussion

### Whole-heart 3D data set at the single-cell scale

Previous studies have performed 3D imaging of the whole mouse heart at single-cell resolution. However, because of the limited imaging range, most of these studies have performed imaging of the embryonic heart to study its development process because the heart is the first-derived functional embryonic organ (32). To the best of our knowledge, only a few complete mature mouse heart data sets have been presented—those imaged through optical imaging techniques, as summarized in Table 1. Knife-edge scanning microscopy (KESM) applied light microscopy to image microtome sectioned tissue on the surface of the knife, achieving a voxel resolution of 0.5 × 0.5 × 5 μm^3^ (33). KESM could locate the spatial distribution of intrinsic cardiac neurons because neurons were stained with cresyl violet and imaged based on structural contrast. However, KESM obtains nonfluorescent labeled structural data, failing to reveal multi-structural spatial relationships. Two-photon tissue cytometry (TPTC) combines two-photon microscopy to perform deep tissue imaging and a milling machine to perform sequential tissue cutting, achieving a voxel resolution of 0.78 × 0.78 × 2.0 μm^3^ (34). TPTC was capable of simultaneously imaging fluorescently labeled cell nuclei and vasculature. However, the point scanning scheme slows down the speed of data acquisition, and the paraffin embedding of the samples prevents labeling with fluorescence proteins. Cardiac light sheet fluorescence microscopy (c-LSFM) owned the advantage of high-speed whole heart imaging through tissue clearing and light-sheet microscope (35). But to cover the whole organ, a long working distance objective with low numerical aperture (0.13) was used, resulting in a low resolution of 4.5 × 4.5 × 18.0 μm^3^.

**Table 1:** Comparison of whole heart optical imaging techniques.

**Imaging techniques**	**KESM**	**TPTC**	**c-LSFM**	**HD-dfMOST**
Imaging resolution (μm^3^)	0.5 ×0.5 ×5	0.78 ×0.78 ×2.0	4.5 ×4.5 ×18.0	0.32 ×0.32 ×1.0
Fluorescence compatibility	N	Y	Y	Y
Observing structures	Cardiac neurons	Cell nuclei and vasculature	Myocardial fiber	Cardiomyocytes and vasculature
Reference	(33)	(34)	(35)	This study

This is the first study to present a set of fluorescently labeled myocardial and vascular heart-wide 3D data for a given mature mouse. We presented individual fine cardiac structures at a single myocyte and capillary level at a submicron voxel imaging resolution of 0.32 × 0.32 × 1.0 μm^3^ (Figures 2, 3). Moreover, we visualized these structures simultaneously to reveal their spatial correlation or locate one structure through the other (Figure 4). Moreover, we analyzed detailed geometric features in both outer and inner views through subsequent 3D data reconstruction (Figure 5).

### Biomedical perspectives

The pipeline established in this study can be used for examining heart-wide 3D fine structures. A global heart-specific coordinate system is required to define the location and orientation of specific structures (29). Here, for the system to be specific to the heart, the coordinate system should be built on the basis of the cardiac anatomy, and the characteristic anatomy of the whole heart should be considered for global imaging. Our pipeline can build such a coordinate system. By using a proper structural labeling method, we can set one channel to image architectural data, such as cardiomyocytes, and build a coordinate system based on three non-coplanar feature points, namely the apex of the heart, centroids of the aorta, and centroids of the descending aorta. In addition, we can set other channels to image target structures such as vessels and neurons. Similar methods have been developed to depict brain-wide connections, including the vascular network and neural connection, through a brain-wide positioning system (19, 21). Thus, a similar approach can be used in heart studies.

The single-cell-resolution heart-wide data set obtained using this pipeline can serve as the basis for heart disease research. For instance, by comparing the structural difference between the normal and diseased heart at the single-cell level, lesions in the whole heart can be precisely located (36). Furthermore, examining the data sets of diseased hearts in different stages of a disease may help understand lesion development in the disease (37). A combination of knowledge derived from these data sets and clinical imaging findings may guide the selection of medication and surgical treatment (38).

### Limitations

We aimed to demonstrate the feasibility and effectiveness of the proposed pipeline; thus, only two cardiac structures, namely the cardiovascular and myocardium, are presented. However, multicolor imaging is not technically difficult. By using proper specific labeling techniques, additional types of cardiac structures, such as collagen and cardiac neurons, can be imaged within a given mouse heart, enabling the study of the whole cardiac structure and heart-wide multistructural linkages. Here we imaged a cardiac slice stained by PI and DAPI solutions under a commercial microscope (Zeiss Axio Observer A1; Zeiss, Germany), and the result is shown in Supplementary Figure 1. This result help demonstrates that multi-color options are feasible. Moreover, only a mature mouse model was used in this study. However, this pipeline can be used for imaging the primate and human heart after appropriate modifications (39).

In this study, we did not perform a large-scale quantitative analysis of cardiac data because cardiac structures are considerably different from brain structures in terms of geometrical dimensions and structural features. For example, neural cells are approximately spherical, whereas cardiomyocytes are approximately cylindrical. Because of this difference, the previously established automated analysis methods, such as 3D BrainCV and NeuroGPS-Tree, could not be fully used in this work (40, 41). Most of the 3D reconstruction results obtained in this study were based on semiautomatic image segmentation, resulting in a considerable workload. To demonstrate the potential of the single-cell-resolution heart-wide data for cell measurement, we segmented a myocardial fiber and five nuclei from Figure 2D, and the result is shown in Supplementary Figure 2. The quantitative statistics of the five nuclei volume is 87.2 ± 6.1 μm. In the future, we will be focusing on developing automated cardiac quantitative analysis tools.

## Data availability statement

The original contributions presented in the study are included in the article/Supplementary materials, further inquiries can be directed to the corresponding author/s.

## Ethics statement

The animal study was reviewed and approved by Institutional Animal Ethics Committee of Huazhong University of Science and Technology.

## Author contributions

QL, HG, JY, JC, and XY conceived and designed the study. WS, YZ, JJ, and JC performed the experiments and data analysis. GL and SC performed the whole-heart data acquisition. HG, JC, and XY prepared the figures and wrote the manuscript. All authors contributed to the article and approved the submitted version.

## Funding

This work was financially supported by the National Natural Science Foundation of China projects (Nos. 81827901, 82102235, and 81871082), Hubei Provincial Natural Science Foundation of China (No. 2020CFB129), and the Director Fund of the Wuhan National Laboratory for Optoelectronics (WNLO).

## Conflict of interest

The authors declare that the research was conducted in the absence of any commercial or financial relationships that could be construed as a potential conflict of interest.

## Publisher's note

All claims expressed in this article are solely those of the authors and do not necessarily represent those of their affiliated organizations, or those of the publisher, the editors and the reviewers. Any product that may be evaluated in this article, or claim that may be made by its manufacturer, is not guaranteed or endorsed by the publisher.

## Supplementary material

Supplementary Figure 1 Image of a cardiac slice stained by PI and DAPI fluorescent dyes. Scale bars: 50 μm.

Supplementary Figure 2 Segment a myocardial fiber and five nuclei from Figure 2D and quantify the volume of these cardiomyocyte nuclei.

Supplementary Video 1 Rendered myocardial data shows the morphology and orientation of myocardial assembly.

Supplementary Video 2 Rendered cardiovascular data shows the complex three-dimensional spatial winding of capillaries.

Supplementary Video 3 Rendered co-localized myocardial and vascular data shows spatial distribution of myocardial and vascular structures.
