# Supplementary figures and images for: Three-dimensional visualization of heart-wide myocardial architecture and vascular network simultaneously at single-cell resolution
Source: Front Cardiovasc Med. 2022 Aug 4;9:945198. doi: 10.3389/fcvm.2022.945198 (PMC9386161; doi:10.3389/fcvm.2022.945198)

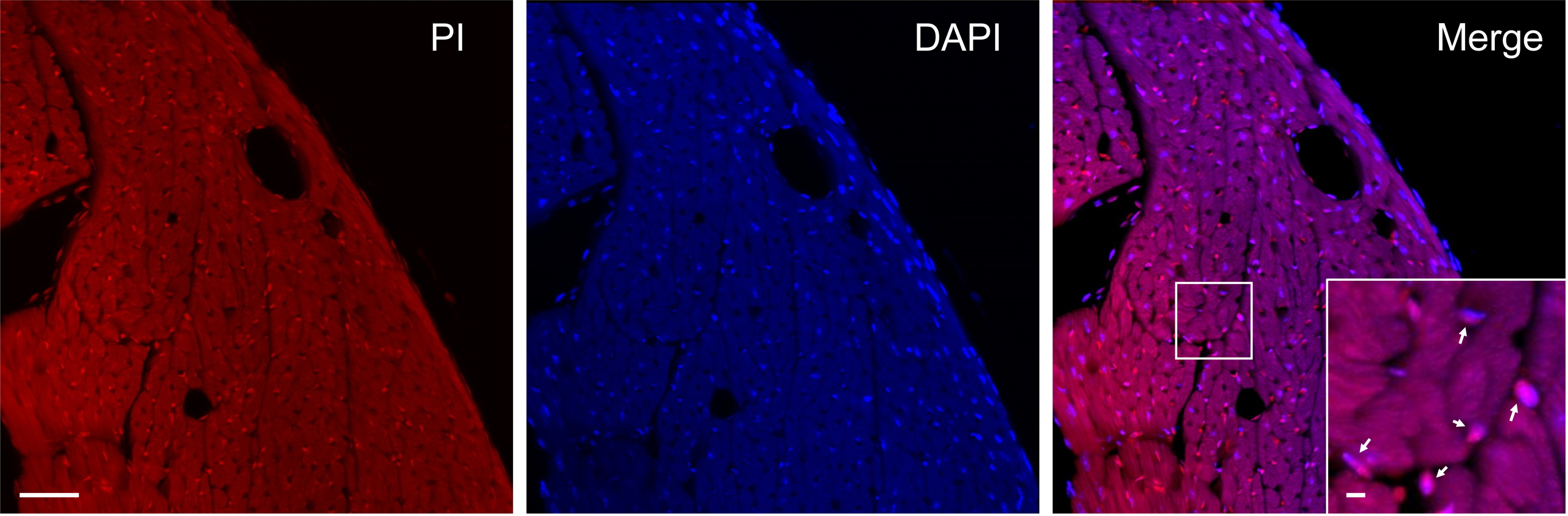

Supplement: Supplementary Figure 1 — Image of a cardiac slice stained by PI and DAPI fluorescent dyes. Scale bars: 50 μm. [file Image_1.jpg]

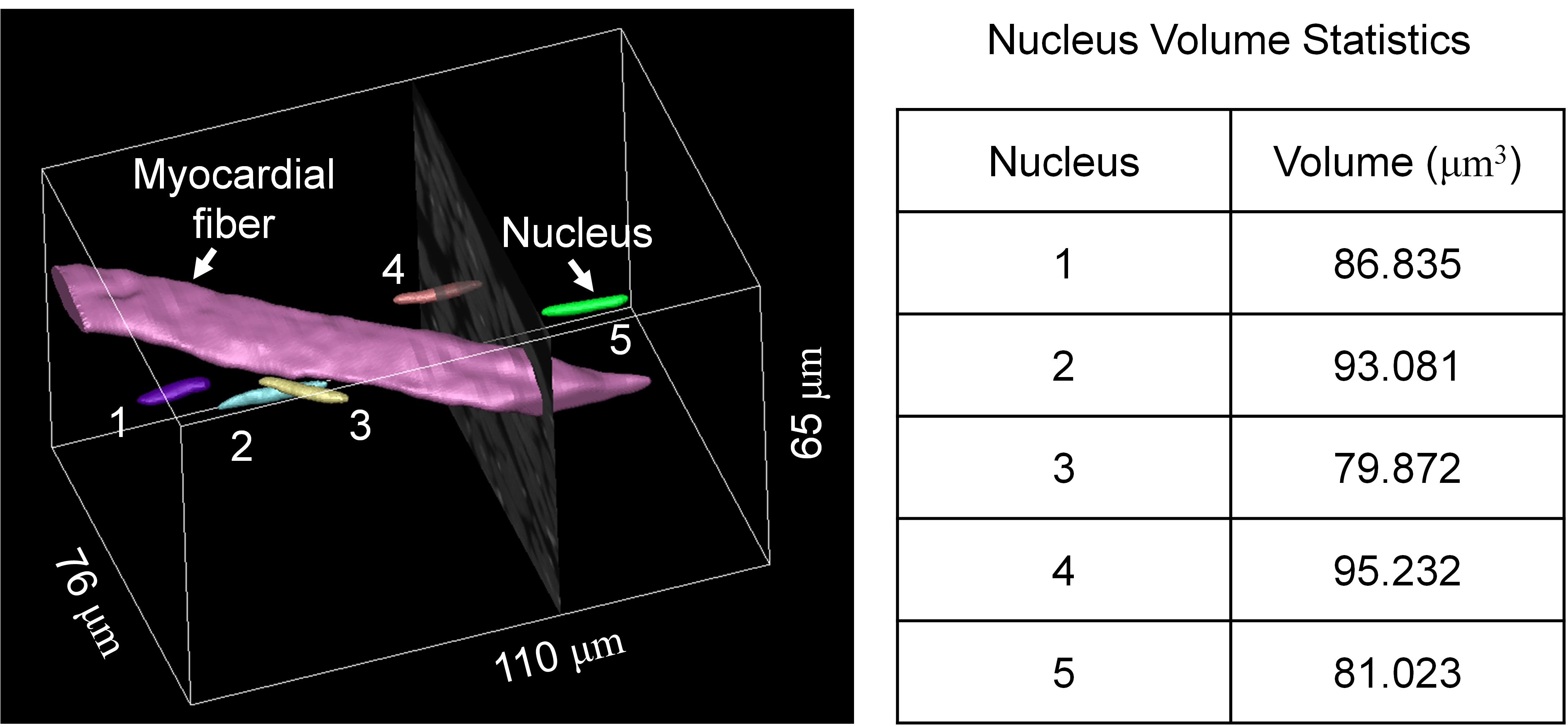

Supplement: Supplementary Figure 2 — Segment a myocardial fiber and five nuclei from Figure 2D and quantify the volume of these cardiomyocyte nuclei. [file Image_2.jpg]
